# Combining sequence-based prediction methods and circular dichroism and infrared spectroscopic data to improve protein secondary structure determinations

**DOI:** 10.1186/1471-2105-9-24

**Published:** 2008-01-15

**Authors:** Jonathan G Lees, Robert W Janes

**Affiliations:** 1 School of Biological and Chemical Sciences, Queen Mary, University of London, London E1 4NS, UK; 2 Department of Biochemistry and Molecular Biology, University College London, University of London, London, WC1E 6BT, UK

## Abstract

**Background:**

A number of sequence-based methods exist for protein secondary structure prediction. Protein secondary structures can also be determined experimentally from circular dichroism, and infrared spectroscopic data using empirical analysis methods. It has been proposed that comparable accuracy can be obtained from sequence-based predictions as from these biophysical measurements. Here we have examined the secondary structure determination accuracies of sequence prediction methods with the empirically determined values from the spectroscopic data on datasets of proteins for which both crystal structures and spectroscopic data are available.

**Results:**

In this study we show that the sequence prediction methods have accuracies nearly comparable to those of spectroscopic methods. However, we also demonstrate that combining the spectroscopic and sequences techniques produces significant overall improvements in secondary structure determinations. In addition, combining the extra information content available from synchrotron radiation circular dichroism data with sequence methods also shows improvements.

**Conclusion:**

Combining sequence prediction with experimentally determined spectroscopic methods for protein secondary structure content significantly enhances the accuracy of the overall results obtained.

## Background

With the growing availability of a large number of new genome sequences, there is an ongoing interest in the structures of the proteins represented by the open reading frames in those genomes. Although there has been a growth in the number of crystal structures of proteins as the result of Structural Protemics programmes worldwide, their production has not kept pace with the sequencing effort. Furthermore, such programmes often produce small amounts of any given protein which are not enough for crystal structure analysis. However, these quantities are often sufficient for other biophysical studies.

Modern sequence-based prediction methods can provide information on the secondary structure content of these proteins without the need for producing any protein. Recent years have seen large improvements in the per-residue secondary structure prediction from sequence [1,2], although they are still limited in their accuracies, especially when applied to certain classes of protein, most notably those with high β-sheet content.

Circular dichroism (CD) and Fourier transform infrared (FTIR) spectroscopies are commonly used techniques for secondary structure content determination, and these methods have also shown great advances in their accuracies [3], with the development of new reference data bases [4] and empirical methods [5-7]. The spectral data collected by these methods requires a relatively small amount of pure protein and can be obtained rapidly [8-11].

The pioneering work of Chou in predicting secondary structure from sequence was through an elaborate and elegant covariance matrix approach [12]. In this study we have examined the performance of sequence based prediction methods using a neural network methodology, versus the experimentally-determined methods of CD and FTIR for assessing secondary structure content. In order to do this, we have used existing reference data sets available in the literature along with established analytical methods and demonstrated the synergies between the different strategies thereby proposing a new combined approach which will greatly improve the results obtained over any single technique that may find use in Structural Proteomics and other structural biology studies.

## Results

### Secondary Structure Predictions from Sequences

Reported correlation coefficients for secondary structure content prediction by CD are typically in the range of *r *= 0.92–0.97 for α-helix (H), *r *= 0.80–0.90 for β-sheet (E) [4,6]. The reported values vary because of differences in the reference dataset, secondary structure assignment and prediction algorithms used. The secondary structure content prediction for sequence prediction methods is generally not reported after publication of the crystal structure. The current highest reported secondary structure content prediction by sequence in the literature is *r *= 0.92 for α-helix and *r *= 0.81 for β-sheet [13]. More recent methods have their performances available on the EVA website [14]. However, currently *r *for secondary structure content prediction is not reported on this web-site.

The per-residue Q3 score of the neural network method is 80.3%. This is in the range of that currently found for the best methods available [15]. The various performance parameters show that the best prediction is for α-helix with slightly lower per-residue prediction accuracy for β-sheet (Table 1). The overall secondary structure content prediction parameters (*r *and δ) are nearly (but not quite in the case of β-sheet content) comparable with those obtained from CD and FTIR methods [4]. An important feature of the sequence prediction methods is that for each residue prediction a corresponding reliability index is obtained. In order to establish if this was useful for assessing the overall reliability of the secondary structure prediction of a protein, a net reliability index was obtained by taking the average reliability index for each of the residues in a protein. We find that there is a weak correlation (*r *= -0.39) between the *abs *error of the secondary structure content prediction and the net reliability index of a protein. The correlation coefficient between the *abs *of secondary structure prediction and the per protein Q2 score is -0.70 and -0.69 for α-helix and β-sheet, respectively. Hence a method with very high secondary structure content prediction accuracy would provide a measure of reliability to a sequence-based prediction.

**Table 1:** GSEQ dataset 3-fold cross-validation.

**Performance measure**	**α-helix (H)**	**β-Sheet (E)**	**Other (G, I, T, B, S, C)**
**Q2 (%)**	89.7	89.2	81.7
**Corr**	0.770	0.676	0.631
**r**	0.939	0.901	0.791
**abs**	0.047	0.047	0.059
δ	0.073	0.069	0.083

Performance parameters from the 3-fold cross-validation of the GSEQ dataset using the neural network sequence-based prediction.

### Secondary Structure Content Prediction

Cross-validation of the RASP46 data set shows that the CD data gives slightly better prediction accuracy than the FTIR data (Table 2). Combining CD and FTIR spectra into composite CD/FTIR spectra gives similar large improvements to that shown previously [16], relative to either of the CD or FTIR methods used separately. The sequence-based prediction for this data set produced better results than the combined spectroscopic methods for helical components but poorer results for sheet components. Combining sequence-based with either type of spectroscopic data improved the sheet content (especially so if CD data were used), but had little effect on the already good helix predictions. A combination of both CD and FTIR data with sequence prediction produced the best overall prediction.

**Table 2:** RASP46 dataset cross-validation.

**Method**	**α-helix (H)**		**β-Sheet (E)**		**Other (G, I, T, B, S, C)**	
	
	**r**	**δ**	**r**	**δ**	**r**	**δ**
**CD**	0.944	0.074	0.916	0.067	0.819	0.077
**FTIR**	0.940	0.076	0.900	0.076	0.740	0.070
**CD_FTIR**	0.955	0.066	0.938	0.060	0.858	0.068
**SEQ**	0.969	0.058	0.917	0.072	0.860	0.079
**CD+SEQ**	0.970	0.055	0.956	0.056	**0.895**	**0.060**
**FTIR+SEQ**	0.974	0.053	0.939	0.062	0.846	0.072
**CD_FTIR+ SEQ**	**0.976**	**0.051**	**0.957**	**0.053**	0.893	0.060

Cross-validation prediction accuracy of the secondary structure content prediction of proteins in the RASP46 dataset. The performance parameters from CD, FTIR, sequence prediction (SEQ), and consensus methods are shown. The best performance parameters for each secondary structural type are shown in bold.

Cross-validation of the SP175 data (Table 3) showed that as previously demonstrated [4] SRCD spectra, which have a higher information content due to additional transitions being measured, produce better results than cCD spectra. In the case of helix content, the experimental- and sequence-based results are very similar, but the combined methods improved all three categories of secondary structure significantly, resulting in a very high correlation with the crystal structures. The RASP46 and SP175 datasets had Q3_Seq _scores of 80.4% and 80.3%, respectively, which are similar to that found from the 3-fold cross-validation of the GSEQ dataset.

**Table 3:** SP175 dataset cross-validation.

**Method**	**α-helix (H)**		**β-Sheet (E)**		**Other (G, I, T, B, S, C)**	
	
	**r**	**δ**	**r**	**δ**	**r**	**δ**
**CD**	0.970	0.053	0.919	0.063	0.787	0.065
**SEQ**	0.972	0.052	0.918	0.068	0.864	0.070
**CD+SEQ**	**0.985**	**0.040**	**0.950**	**0.054**	**0.894**	**0.050**

Cross-validation prediction accuracy of the secondary structure content prediction of proteins in the SP175 dataset. The performance parameters from CD, sequence based (SEQ), and consensus methods are shown. The best performance parameters for each secondary structural type are shown in bold.

### Spectroscopic Identification of Poor Sequence-Based Predictions

Another question that can be addressed is whether secondary structure content measurements can be used to aid with the overall accuracy of a sequence-based prediction method. We see a negative correlation between the Q2 scores and the *abs *difference between the content prediction from sequence and spectroscopy with *r *= -0.3 for α-helix and *r *= -0.5 for β-sheet from the RASP46 dataset and *r *= -0.4 for α-helix and *r *= -0.4 for β-sheet from the SP175 dataset.

**Figure 1:**
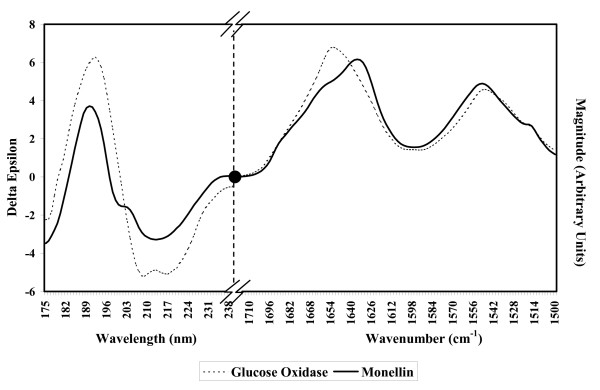
**Composite FTIR and CD spectra**. The CD spectra are in Δε units (y-axis) with wavelengths in nm (x-axis). The FTIR spectra are in arbitrary intensity units scaled as described in the methods (y-axis), with the wavenumbers in cm^-1 ^(x-axis). A pair of hashed lines indicates the discontinuity point for the axes, and the black circle indicates the join between the CD and FTIR spectra.

Examining the individual per protein Q3 scores from sequence prediction of the RASP46 datasets and the SP175 datasets we see that monellin has the poorest score for both datasets, with a Q3 score of 45.7%. The sequence prediction of secondary structure content for monellin produces 27% α-helix and 20% β-sheet, as compared to the 17% α-helix and 51% β-sheet found in the crystal structure. The CD and FTIR spectra (Figure 1) clearly show monellin to have characteristics of a predominantly β-sheet-containing protein in greater quantity than that predicted from sequence. A composite CD and FTIR spectrum of glucose oxidase, a protein whose crystal structure (1cf3) contains 27% α-helix and 20% β-sheet (a content very close to the sequence-predicted values for monellin) is plotted for comparison. The NN prediction using the CD and FTIR data in RASP46 dataset (with monellin removed) plus the sequence information gives a prediction of 12% α-helix and 43% β-sheet content, a result much closer to the actual values.

## Discussion and Conclusion

The results above show that for the proteins in the RASP46 and SP175 reference datasets, a similar secondary structure content accuracy is obtained from the sequence prediction methods as from the experimentally-based methods. Combining the sequence and the cCD and FTIR approaches results in a significantly improved accuracy, especially for β-sheet-rich proteins. For SRCD data, combining this with the sequence prediction method, similarly there is a significant improvement over both these approaches when evaluated in isolation. Notably there is more information content available from the SRCD extended wavelength data which leads to improved accuracy in determining individual secondary structure components [17]. Restricting the defined secondary structures to the three components, as is common practice for FTIR and secondary structure predictions, imposes identical limitation requirements on the SRCD data although many more components than these can be distinguished. The considerable improvement in α-helix over β-sheet and 'Other' reflects that this secondary structure component still provides the dominant spectral characteristics to SRCD spectra. Were FTIR data to be collected for the proteins of the SP175 dataset then this would most likely lead to further significant improvements when combined with the SRCD data and sequence prediction methods. In future, other methods such as Raman Optical Activity [18] could be incorporated into these combinations which would likely result in a further overall improvement.

There are many Structural Proteomics programmes producing proteins for crystal structure investigations. Of note, a number of these proteins prove difficult to obtain in the amounts necessary for such investigations. However, in these cases spectroscopic methods such as SRCD, notable because of the small amounts of material needed to obtain spectra, [8,9] in combination with secondary structure sequence prediction methods could well provide significant insight into the protein structure. A further benefit arises from such a combined approach. Sequence-based methods can sometimes generate poor predictions for protein secondary structure content, which can go unrecognised and therefore lead to further inaccuracies as a result. The reliability of the prediction results can be tested when in combination with the experimental-based spectroscopic methods, thus providing an improved measure of reliability.

There is evidence that from SRCD data the higher information content enables fold information to be obtained from the data [ref. [17] and Wallace, personal communication]. Such fold information combined with a method for tertiary structure prediction such as Threader prediction techniques [15,19] could offer a novel approach to modeling protein structures, potentially with an improved accuracy. The combination of sequence prediction methods with experimental spectroscopically-determined methods for secondary structure content offer a valuable addition for gaining information about protein structure, and maybe potentially an insight into function as a result.

## Methods

### Spectroscopic Datasets (RASP46 and SP175)

The RASP50 dataset consists of 50 composite CD and FTIR spectra [20]. The spectra were obtained with kind permission of Dr. K. Oberg (MannKind Corporation). The CD spectra are in the range of 185–240 nm. The FTIR spectra were in the range of 1720-1500 cm^-1 ^at 1 cm^-1 ^intervals. Combining the two different methods was previously shown to give improvements in performance of secondary structure determination [16]. The spectra were water baseline-subtracted but not side chain-subtracted, as described in the original publication [20]. The area of each of the FTIR spectra was scaled to a total intensity of 663, so that the sum of the integrated areas under the CD and FTIR curves were roughly equal in all cases, ensuring that neither the CD nor FTIR information dominated in the secondary structure predictions. The RASP50 dataset was modified to produce the RASP46 data set, by removing four spectra as follows: The spectrum of rennin appeared to have a disordered spectrum and so was removed. The spectrum of ricin was removed because of uncertainties establishing a match between the PDB file and the protein sequence provided from the bioscience supplier [20]. The spectrum of α-hemolysin was excluded since this membrane protein would be unsuitable for the sequence prediction methods implemented in this study which were designed for soluble proteins. The spectrum of insulin was removed because the 20 amino acid chain B sequence of the PDB file 1trz would not produce an output from the PSI-BLAST [21] program.

The SP175 dataset [4] was a synchrotron radiation circular dichroism (SRCD) dataset consisting of 72 spectra in the range of 175–240 nm. This dataset was used because it has been suggested that the higher information content in SRCD spectra relative to conventional CD (cCD) spectra, will produce more accurate secondary structure analyses [5,17]. From this dataset the spectrum of jacalin was removed because the 18 residue B chain of PDB file 1ku8 would not produce a successful PSI-BLAST output. The CD spectra of pectate-lyase C and ferredoxin were not available when the current study was carried out, so these were also not included in the SP175 dataset used in this study. The CD spectra from both the RASP46 and SP175 datasets were expressed in Δε units.

### Neural Network Derived Sequence Dataset (GSEQ)

The PISCES [22] server uses a combination of PSI-BLAST and structure-based alignments to determine sequence identities and was used to provide a non-redundant dataset for training and testing the sequence-to-secondary structure neural network (NN) predictor as follows: The 25% sequence identity and ≤ 2.5 Å resolution cutoff dataset was downloaded from the PISCES server [22]. Any PDB files containing membrane proteins were removed from the dataset. The sequence database used for PSI-BLAST alignments was the UNIPROT_100 sequence dataset [23]. This was filtered to remove low-complexity regions, transmembrane regions and coiled coil segments using the pfilt [24] algorithm. Position specific scoring matrix (PSSM) profiles were generated for the PISCES dataset sequences by running 3-iterations of PSI-BLAST with the -h option, the threshold for sequence inclusion in the next iteration, set to 0.001. It was noted that the PSI-BLAST algorithm should be run with the -v option, the upper limit value for the number of sequence matches in any run, above the default value of 500 since many proteins gave more than this number of matches. Possible homologues between the PISCES-derived dataset and the spectroscopy datasets were identified by running 5 iterations of PSI-BLAST on the RASP46 and SP175 proteins sequences using the UNIREF100 sequence dataset [23]. After this, any proteins were removed from the PISCES-derived dataset if they had E-value scores ≤ 5.0 with any proteins in the RASP46 or SP175 datasets. Finally, any proteins of sequence length < 30 amino acids were removed from the dataset. The final dataset, which we designate GSEQ, contained 2984 protein chains. The PSSM profiles were scaled to be between 0 and 1 by the standard logistic function. An extra input for each residue was used to indicate if the central residue was passed the N- or C-terminus.

The secondary structure assignment scheme applied was that of α-helix (H), β-sheet and other (G, I, B, S, T, C), using the designation provided in the DSSP output file [25]. This secondary structure assignment has previously been shown to be appropriate for both sequence based [15] and CD or FTIR spectroscopic prediction methods [5,16].

### Neural Network Architecture and Training

The PSI-PRED algorithm creates a simple NN architecture that has been shown to be amongst the best methods for secondary structure prediction from sequence [14]. The NNs constructed in this work had identical architectures to that used in the original PSI-PRED paper [15]. They consisted of 15 × 21 input, and 75 hidden neurons (units in the original work [15]) in the sequence-to-structure neural network and, separately, 4 × 15 inputs and 60 hidden neurons (units) in the structure-to-structure network. The training parameters for online back-propagation were also maintained at the same values (momentum = 0.9, learning rate = 0.005) as in the original PSI-PRED publication. The training parameters and network architecture were not optimised since the main purpose of this paper was to reveal relative differences and potential synergies between sequence-based and spectroscopic-based methods under similar conditions.

Training and testing were carried out using a 3-fold cross-validation of the network. Initially, during training 10% of the training data was kept aside as the validation set and not included in the training. Training was stopped when the performance of the validation set began to degrade relative to the training set. The GSEQ dataset was then used to assess the performance of the network by 3-fold cross validation as given in Table 1. The test sets in Tables 2 and 3, RASP46 and SP175, respectively, contained none of the training set of proteins.

### Assessment of the Prediction Accuracy

Secondary structure content prediction methods are typically measured using the widely reported Pearsons correlation coefficient (*r*). Values of *r *range between +1 and -1 representing perfect positive and negative correlation respectively. Additionally either the root mean squared deviation (*δ*), or the absolute deviation (*abs*) are reported. When judging the performance of a method, high values of *r *and low values of *δ *indicate good performance. Corr is the Mathews correlation coefficient [26].

Assessment of the prediction accuracy on the RASP46 and SP175 datasets was carried out using the SIMPLS algorithm [27] combined with zeroing negative fractions and rescaling to 100%. The PDB files and corresponding secondary structure contents for the datasets were those described in the original publications which produced the datasets. The performance parameters for these datasets were assessed using full cross-validation. Sequence-to-structure predictions on the sequences were carried out by taking the average of the prediction of the three networks produced from the 3-fold cross-validation on the corresponding proteins in each of the experimental datasets.

## Authors' contributions

JGL did the calculations and RWJ supervised this work. Both authors participated in the writing, and have read and approved of the manuscript. Neither of the authors have any competing financial or other interests in relation to this work.
